# Palliative Treatment of Leptomeningeal Carcinomatosis From Renal Cell Carcinoma With Local CyberKnife Radiotherapy and Systemic Pazopanib Therapy: A Case Report

**DOI:** 10.7759/cureus.54025

**Published:** 2024-02-11

**Authors:** Shinichiro Mizumatsu, Kenichi Wakabayashi, Yasuhiro Terashima

**Affiliations:** 1 Cyberknife Center, Aoyama General Hospital, Toyokawa, JPN; 2 Department of Neurosurgery, Toyohashi Municipal Hospital, Toyohashi, JPN; 3 Department of Urology, Toyohashi Municipal Hospital, Toyohashi, JPN

**Keywords:** choroid plexus metastasis, internal auditory canal metastasis, stereotactic radiotherapy (srt), palliative treatment, molecular-targeted therapy, cyberknife radiotherapy, pazopanib, renal cell carcinoma (rcc), leptomeningeal carcinomatosis

## Abstract

Leptomeningeal carcinomatosis (LMC) from renal cell carcinoma (RCC) is rare. There is no established treatment strategy for LMC, and the prognosis is extremely poor. We describe a case of LMC from RCC treated with local CyberKnife radiotherapy (CKR) and systemic therapy with pazopanib. The patient was a 63-year-old man with brain metastases from right RCC. Surgery and CKR were performed for the brain metastases, and the lesions were subsequently controlled. The patient developed isolated lesions in the pituitary stalk, right internal auditory canal, left ventricular choroid plexus (CP), left facial nerve, and medulla oblongata after the surgery and CKR for brain metastases. We diagnosed LMC and treated the patient with systemic therapy with pazopanib. We performed local therapy with CKR for lesions of the pituitary stalk, right internal auditory canal, left facial nerve, and medulla oblongata. The CP lesion was not treated with CKR because the lesion tended to shrink after systemic therapy with pazopanib. There were no symptoms due to LMC until the end of life and no adverse events due to CKR. Ten years and five months after the nephrectomy for RCC, one year and four months after the initial CKR for brain metastases, and nine months after the diagnosis of LMC, the patient died due to pleural effusion from lung metastases. Our case suggests that CKR combined with pazopanib may be effective as a palliative treatment for LMC from RCC.

## Introduction

Leptomeningeal carcinomatosis (LMC) is a rare late complication that occurs in 5-8% of solid tumors and 5-15% of hematological cancers and has a poor prognosis despite advances in treatment. If untreated, the time from diagnosis to death is approximately 4-6 weeks, and overall survival with treatment is approximately 2-4 months [1]. LMC causes a variety of symptoms and requires palliative treatment. LMC due to renal cell carcinoma (RCC) is very rare, with only six cases reported to the best of our knowledge [2-7]. Conventionally, whole-brain radiotherapy (WBRT) is often used as radiotherapy for LMC. WBRT is usually administered at 30-40 Gy divided by 2-3 Gy per fraction. Craniospinal radiotherapy is necessary to eradicate the tumor but is associated with very high systemic and central nervous system toxicity and carries the risk of myelosuppression and other complications. RCC has low radiosensitivity in conventional radiation therapy (CRT). Compared to CRT, stereotactic radiotherapy (SRT) reduces the risk of damage to surrounding organs and delivers more intensive radiation in a shorter time [8]. CyberKnife® (CK) (Accuray Incorporated, Sunnyvale, CA, USA) [9] is a type of image-guided SRT system that delivers high-dose radiation to the tumor with precision. Because SRT is a local therapy, it should be combined with systemic therapy that can control the spread of LMC. Pazopanib is a molecular-targeted therapy with proven efficacy against RCC [10]. There are no reports of the use of pazopanib for LMC from RCC. We report a case of LMC from RCC treated with local radiotherapy with CK and systemic therapy with pazopanib.

## Case presentation

**Figure 1:**
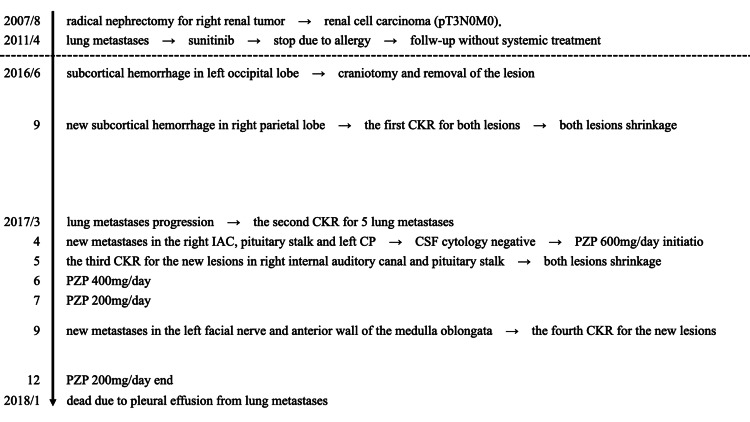
Clinical course. CKR: CyberKnife radiotherapy; IAC: Internal auditory canal; CP: Choroid plexus; PZP: Pazopanib; CSF: Cerebrospinal fluid.

The clinical course is shown in Figure 1.

The patient, a 63-year-old male, underwent a radical nephrectomy for a right kidney tumor at the age of 54. The histological diagnosis was clear cell RCC (pT3bN0M0). At age 58, the patient developed lung metastases and was started on systemic therapy with sunitinib. However, the treatment was soon discontinued due to a severe allergic reaction. The patient was then followed up without systemic therapy.

At age 63 (8 years and 10 months after the surgery for RCC), the patient presented with dizziness, nausea, and right homonymous hemianopsia. CT and MRI demonstrated a subcortical hemorrhage in the left occipital lobe. The lesion was treated with craniotomy and resection.

**Figure 2:**
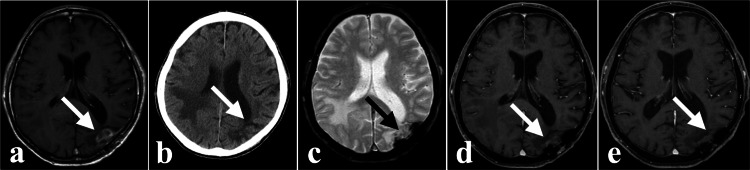
Time course of the left occipital lobe lesion. (a), (b), and (c) are pre-CKR images taken 3 months after brain surgery. (a), (d), and (e) show enhanced MRIs. (a) Enhanced lesion (indicated by the white arrow). (b) No rebleeding visible on plain CT (indicated by the white arrow). (c) No rebleeding visible on T2*WI (indicated by the black arrow). (d) Shrinkage of the enhanced lesion observed 1 month after CKR (indicated by the white arrow). (e) No lesion recurrence observed 12 months after CKR (indicated by the white arrow). CKR: CyberKnife radiotherapy; T2*WI: T2*-weighted image.

**Figure 3:**
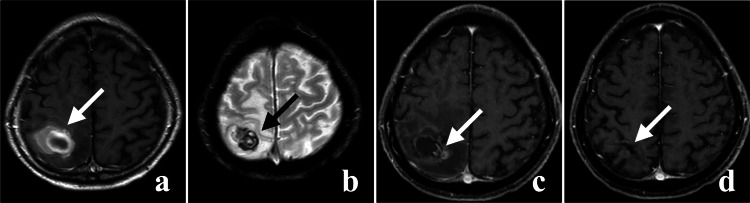
Time course of the right parietal lobe lesion. (a) and (b) are pre-CKR images taken 2 months after subcortical hemorrhage. (a), (c), and (d) show enhanced MRIs. (a) Enhanced lesion with peripheral contrast leakage (indicated by the white arrow). (b) Heterogeneous low-signal nodule on T2*WI (indicated by the black arrow). (c) Shrinkage of the enhanced lesion observed 1 month after CKR (indicated by the white arrow). (d) No lesion recurrence observed 12 months after CKR (indicated by the white arrow). CKR: CyberKnife radiotherapy; T2*WI: T2*-weighted image.

One month after the brain surgery, the patient presented with left lower extremity paralysis and generalized rigid epileptic seizures. A new subcortical hemorrhage occurred in the right parietal lobe, but the patient was treated conservatively. Furthermore, one month later, another epileptic seizure occurred, and the patient was admitted to another hospital for an emergency. The left occipital lobe lesion and the right parietal lobe lesion were diagnosed as brain metastases from RCC because MRI showed contrast enhancement (Figures 2a, 3a) and ^18^F-fluorodeoxyglucose positron emission tomography (FDG-PET)/CT showed uptake.

The patient was referred to our hospital for CK radiotherapy (CKR) for brain metastases.

**Figure 4:**
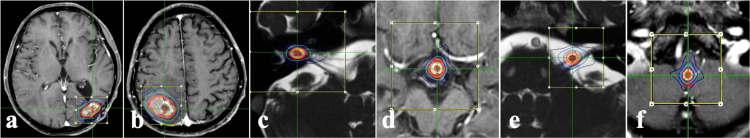
CKR plan using MultiPlan. These are the dose distributions in MultiPlan® (Accuray Incorporated, Sunnyvale, USA): (a) The left occipital lesion. (b) The right parietal lesion. (c) The right internal auditory canal lesion. (d) The pituitary stalk lesion. (e) The left facial nerve lesion. (f) The medullary lesion. CKR: CyberKnife radiotherapy.

Upon admission, the patient had only mild weakness in the left lower extremity. CT and MRI before CKR showed no rebleeding in the left occipital lobe lesion (Figures 2b, 2c) and tumor hemorrhage in the right parietal lobe (Figures 3b). We performed CKR on the left occipital lobe lesion (target volume 2.78 ml, marginal dose 36.0 Gy, maximum dose 64.1 Gy, 5 fractions, 7 days) (Figure 4a) and the right parietal lobe lesion (target volume 16.16 ml, marginal dose 35.2 Gy, maximum dose 67.4 Gy, 5 fractions, 7 days) (Figure 4b, Table 1).

**Table 1:** CKR treatment parameters. CKR: CyberKnife radiotherapy.

CKR for brain lesions	Location	Target volume (ml)	Fractions	Marginal dose (Gy)	Maximum dose (Gy)	Treatment periods (days)
2016 September	Left occipital lobe	2.78	5	36.0	64.1	7
	Right parietal lobe	16.16	5	35.2	67.4	7
2017 May	Right internal canal	0.09	5	25.4	40.6	7
	Pituitary stalk	0.28	5	20.0	43.7	7
2017 September	Left facial nerve	0.09	5	25.2	37.1	7
	Medulla oblongata	0.06	3	25.5	33.6	6

One month after the CKR, the lesions shrank (Figures 2d, 3c). These two lesions were controlled until the end of life (Figures 2e, 3d).

**Figure 5:**
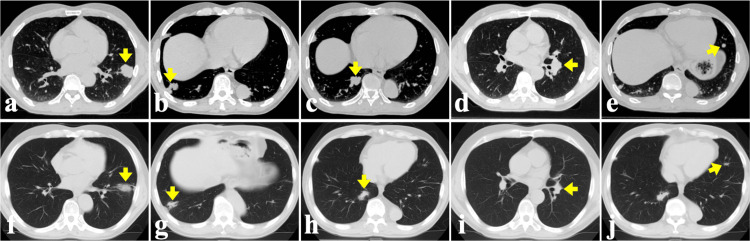
The lung metastases on plain CT before and after the CKR. (a)-(e) Five lung lesions before CKR. (f)-(j) Five lung lesions 6 months after CKR. CKR: CyberKnife radiotherapy.

Six months after the CKR for brain metastases, he was treated with a second CKR for five lung metastases. The five lung metastases were controlled until the end stage (Figure 5).

**Figure 6:**
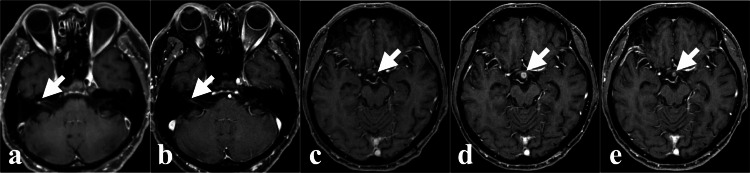
Time course of the right internal auditory canal and pituitary stalk lesions on enhanced axial MRI. (a) and (b) depict the right internal auditory canal lesion. (c), (d), and (e) depict the pituitary stalk lesion. (a) Enhanced lesion at CKR (indicated by the white arrow). (b) No lesion four months after CKR (indicated by the white arrow). (c) Enhanced pituitary stalk two months before CKR (indicated by the white arrow). (d) Increased enhancement of the lesion at CKR (indicated by the white arrow). (e) Shrinkage of the enhanced lesion four months after CKR (indicated by the white arrow). CKR: CyberKnife radiotherapy.

One month after the CKR for the lung metastases, two new solitary lesions developed in the right internal auditory canal (Figure 6a) and the pituitary stalk (Figures 6c-6d).

**Figure 7:**
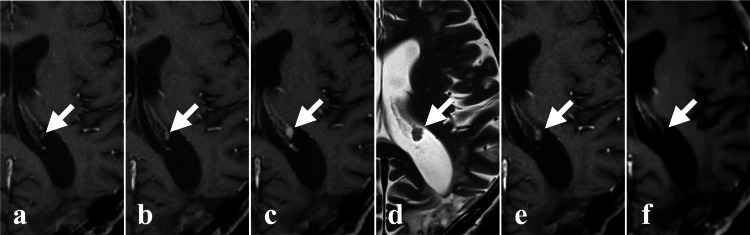
Time course of the choroid plexus lesion in the left lateral ventricle on axial MRI. (a) Lesion is not evident (indicated by the white arrow). (b) Slightly increased enhancement of the lesion 1 month after (a) MRI (indicated by the white arrow). (c) Further increase in the lesion 1 month after (b) MRI (indicated by the white arrow). (d) Further increase in the lesion on T2WI 2 months after (c) MRI (indicated by the white arrow). MRI showing the maximum lesion 2 weeks before PZP starts. (e) Shrinkage of the lesion 3 weeks after (d) MRI (indicated by the white arrow). MRI showing the decreased lesion 6 days after PZP start. (f) Further shrinkage of the lesion 15 weeks after (e) MRI (indicated by the white arrow). MRI showing complete response (CR) of the lesion 16 weeks after PZP start. T2WI: T2-weighted image; CR: Complete response; PZP: Pazopanib.

An increasing nodular contrast-enhanced lesion was also found on the choroid plexus (CP) in the left lateral ventricle (Figures 7a-7d).

The patient had no symptoms associated with the new lesions. Cerebrospinal fluid (CSF) cytology was negative, but we considered LMC. The patient was started on systemic therapy with pazopanib 600 mg/day.

We performed CKR on the right internal auditory canal lesion (target volume 0.09 ml, marginal dose 25.4 Gy, maximum dose 40.6 Gy, 5 fractions, 7 days) (Figure 4c) and the pituitary stalk lesion (target volume 0.28 ml, marginal dose 20.0 Gy, maximum dose 43.7 Gy, 5 fractions, 7 days) (Figure 4d, Table 1). The left lateral ventricular lesion was excluded from the CKR target because it had shrunk on pre-CKR MRI (6 days after pazopanib initiation) (Figure 7e). The CKR-treated lesions showed shrinkage on follow-up MRI (Figures 6b, 6e). The untreated lesion in the left lateral ventricle demonstrated a further reduction in size on follow-up imaging (Figure 7f).

**Figure 8:**
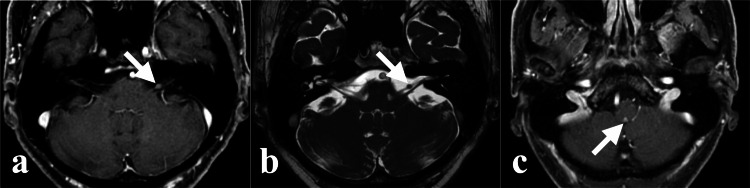
The left facial nerve and medullary lesions on axial MRI before CKR. (a) Enhanced lesion of the left facial nerve (indicated by the white arrow). (b) Nodular lesion of the left facial nerve on heavy T2WI (indicated by the white arrow). (c) Enhancement of the medullary lesion (indicated by the white arrow). CKR: CyberKnife radiotherapy, T2WI: T2 weighted image.

Four months after the prior CKR, two new lesions developed in the left facial nerve (Figures 8a, 8b) and medulla oblongata facing the cerebellomedullary cistern (Figure 8c).

The patient had no symptoms due to the new lesions. We performed CKR on the left facial nerve lesion (target volume 0.09 ml, marginal dose 25.2 Gy, maximum dose 37.1 Gy, 5 fractions, 7 days) (Figure 4e) and the medullary lesion (target volume 0.06 ml, marginal dose 25.5 Gy, maximum dose 33.6 Gy, 3 fractions, 6 days) (Figure 4f, Table 1).

The patient died at age 65 due to pleural effusion from lung metastases 8 years and 6 months after the first treatment for RCC, 19 months after brain surgery, 16 months after the first CKR, and 9 months after the onset of LMC. The patient had been taking pazopanib 200 mg/day for 25 days before death. As far as our follow-up was concerned, spinal MRI showed no evidence of disseminated lesions. The patient had no symptoms related to LMC until the end of his life and no adverse events due to CKR.

## Discussion

LMC from RCC is extremely rare. To the best of our knowledge, only six cases of LMC from RCC have been reported [2-7]. In our case, CSF cytology was negative, but we suspected LMC. Autopsy studies on LMC have reported negative CSF cytology in 50% to 60% of cases [11]. Although CSF cytology has a specificity of 100%, its sensitivity may not be necessarily high. Therefore, a negative CSF cytology does not rule out the diagnosis of LMC. MRI with gadolinium contrast has a sensitivity of 70% and a specificity of 77% to 100% [1]. Because MRI alone has low sensitivity, combining it with CSF cytology is expected to improve diagnostic accuracy. Therefore, in clinically and radiologically suspicious cases, LMC should not be excluded based on negative CSF cytology alone.

We diagnosed LMC because of the continuous occurrence of lesions in direct contact with the CSF. RCC is reported to be the most common primary site of CP metastasis [12-14]. Garrido E et al. reported that three-quarters of intraventricular metastases develop in the lateral ventricle, then respectively in the fourth and third ventricles. The prognosis remains poor due to dissemination via the CSF [14]. However, CP metastases are not always indicative of LMC, as Crisman CM et al. reported that LMC was not observed in 22 CP metastases in 19 patients with RCC [13].

Regardless of whether RCC is the primary lesion, LMC has an extremely poor prognosis with no established treatment. As LMC progresses, various symptoms are expected to occur. Palliative treatment for LMC is necessary in symptomatic cases, and treatment to prevent the development of symptoms of LMC is necessary in asymptomatic cases. WBRT has traditionally been used for LMC but is problematic because it is associated with decreased cognitive function and quality of life.

In our case, when the LMC lesions progressed, we anticipated the development of hearing disturbances, facial paralysis, endocrine and visual dysfunction, hydrocephalus, etc. We treated the patient locally with CKR and systemically with pazopanib because the disseminated lesions were solitary and RCC was not radiosensitive.

In our case, CKR prevented symptoms secondary to LMC without adverse events due to CKR. Compared with CRT, SRT reduces the risk of damage to surrounding organs and allows high doses of radiation to be focused on the lesion in a shorter period. CK is a near real-time image-guided SBRT device with a robotic arm, linear accelerator, and target tracking system [9]. CKR is effective as a local treatment for the prevention or palliation of symptoms. However, radiation therapy is a local therapy and cannot prevent the intrathecal spread of tumor cells. Therefore, it must be combined with systemic therapy such as chemotherapy.

Four molecular-targeted agents have been reported for treating LMC from RCC (Table 2).

**Table 2:** Summary of published cases of molecular targeted therapy for the LMC from RCC. LMC: Leptomeningeal carcinomatosis; RCC: Renal cell carcinoma; PR: Partial response; ND: Not described; PD: Progressive disease; CR: Complete response; CP: Choroid plexus.

Author	Generic name	Therapeutic effect	Result	Cause of death
Ranze O et al., 2007 [3]	Sorafenib	PR	The lesions decreased 10 days after initiation of sorafenib. PR at least 10 weeks.	ND
Dalhaug A et al., 2010 [4]	Sunitinib	PD	After 2 weeks on sunitinib, reduced general condition with fever, leukopenia, and thrombopenia. The patient died 4 months after the LMC diagnosis.	Pneumonia
Chilkulwar A et al., 2014 [6]	Temsirolimus	PD	After 3 courses of temsirolimus treatment, the lower extremity symptoms worsened and it was necessary to treat the hydrocephalus.	ND
Bonomi L et al., 2020 [7]	Nivolumab	CR	CR of LMC and lung metastases. The patient rapidly improved cancer-related symptoms without drug-related toxicity.	ND
Our Case, 2024	Pazopanib	PR - CR	The CP lesion decreased 6 days after initiation of pazopanib. The patient survived 9 months after LMC.	Lung metastases

Efficacy was reported for nivolumab [7] and sorafenib [3], but not for temsirolimus [6] and sunitinib [4]. In particular, nivolumab was reported to lead to complete remission and is expected to be a therapeutic agent when LMC is detected early [7]. In our case, nivolumab was not yet covered by public insurance and could not be used. Pazopanib is a multitarget tyrosine kinase inhibitor of vascular endothelial growth factor receptors, platelet-derived growth factor, and c-Kit, approved by the FDA for advanced RCC [10]. There have been no randomized controlled trials on the use of pazopanib for brain metastases from RCC. Animal studies have shown that it passes the blood-brain barrier [15], but there are no data on humans. Several case reports have indicated that pazopanib is effective against brain metastases from RCC [16-18]. Mojica CV et al. reported that the use of pazopanib for brain metastases from RCC resulted in a 36-month progression-free survival (PFS) but relapsed after drug discontinuation [18]. Hingorani M et al. reported that pazopanib 800 mg/day was started in a patient who had undergone WBRT (30 Gy/10 fractions) for brain metastases after treatment with sunitinib, and the dose was reduced to 600 mg/day after 6 months, resulting in a PFS of 11 months [17]. Jacobs C et al. reported that high-dose pazopanib 1 g/day was effective in a patient who relapsed on standard-dose pazopanib 800 mg/day after WBRT (30 Gy/10 fractions) for brain metastases [16]. There are no reports of pazopanib treatment for LMC from RCC. In our case, the CP lesion in the left ventricle, which had been increasing, shrank on MRI six days after pazopanib initiation. The shrinkage of the lesion in the absence of CKR can be attributed to the effect of pazopanib treatment. Furthermore, pazopanib may have inhibited dissemination, as the number of increased LMC lesions was minimal. In addition, survival from LMC diagnosis was relatively long (nine months), suggesting that pazopanib may have contributed to the prolonged prognosis.

Our case suggests that local radiotherapy with CKR and systemic therapy with pazopanib may be effective as palliative treatment for LMC from RCC. However, due to the small number of reported cases, an effective treatment for LMC from RCC cannot be determined at this time.

## Conclusions

LMC from RCC is rare and often challenging to diagnose early. However, even in the case of a solitary lesion, it is necessary to suspect CSF dissemination, especially if the lesion is in direct contact with the CSF cavity. There is no established treatment for LMC, regardless of whether the primary source is RCC. Our case suggests that CKR may be a viable palliative local treatment option for LMC from RCC. Furthermore, the addition of systemic therapy with pazopanib to CKR may inhibit the spread of CSF dissemination and improve therapeutic efficacy. In the future, the most effective treatment for LMC from RCC will need to be determined by the accumulation of more cases.

## References

[1] Batool A, Kasi A (2023). Leptomeningeal Carcinomatosis. https://www.ncbi.nlm.nih.gov/books/NBK499862/.

[2] Tippin DB, Reeves W, Vogelzang NJ (1999). Diagnosis and treatment of leptomeningeal metastases in a patient with renal carcinoma responding to 5-fluorouracil and gemcitabine. J Urol.

[3] Ranze O, Hofmann E, Distelrath A, Hoeffkes HG (2007). Renal cell cancer presented with leptomeningeal carcinomatosis effectively treated with sorafenib. Onkologie.

[4] Dalhaug A, Haukland E, Nieder C (2010). Leptomeningeal carcinomatosis from renal cell cancer: treatment attempt with radiation and sunitinib (case report). World J Surg Oncol.

[5] Huang JY, Tan SC (2011). Clinical manifestation of RCC leptomeningealcarcinomatosis-A case study. Int J Surg Case Rep.

[6] Chilkulwar A, Pottimutyapu R, Wu F, Padooru KR, Pingali SR, Kassem M (2014). Leptomeningeal carcinomatosis associated with papillary renal cell carcinoma. Ecancermedicalscience.

[7] Bonomi L, Bettini AC, Arnoldi E (2020). Nivolumab efficacy in leptomeningeal metastasis of renal cell carcinoma: a case report. Tumori.

[8] Majeed H, Gupta V (2023). Adverse Effects of Radiation Therapy. https://www.ncbi.nlm.nih.gov/books/NBK563259/.

[9] Adler JR Jr, Chang SD, Murphy MJ, Doty J, Geis P, Hancock SL (1997). The Cyberknife: a frameless robotic system for radiosurgery. Stereotact Funct Neurosurg.

[10] Sternberg CN, Davis ID, Mardiak J (2023). Pazopanib in locally advanced or metastatic renal cell carcinoma: results of a randomized phase III trial. J Clin Oncol.

[11] Glass JP, Melamed M, Chernik NL, Posner JB (1979). Malignant cells in cerebrospinal fluid (CSF): the meaning of a positive CSF cytology. Neurology.

[12] Siomin V, Lin JL, Marko NF (2011). Stereotactic radiosurgical treatment of brain metastases to the choroid plexus. Int J Radiat Oncol Biol Phys.

[13] Crisman CM, Patel AR, Winston G, Brennan CW, Tabar V, Moss NS (2020). Clinical outcomes in patients with renal cell carcinoma metastases to the choroid plexus. World Neurosurg.

[14] Garrido E, Alqahtani K, Lozouet M, Derrey S, Gilard V (2023). Metastasis of the choroid plexuses: a systematic review of the literature and case illustration. Neurochirurgie.

[15] Minocha M, Khurana V, Mitra AK (2012). Determination of pazopanib (GW-786034) in mouse plasma and brain tissue by liquid chromatography-tandem mass spectrometry (LC/MS-MS). J Chromatogr B Analyt Technol Biomed Life Sci.

[16] Jacobs C, Kim DW, Straka C, Timmerman RD, Brugarolas J (2013). Prolonged survival of a patient with papillary renal cell carcinoma and brain metastases using pazopanib. J Clin Oncol.

[17] Hingorani M, Dixit S, Maraveyas A (2014). Pazopanib-induced regression of brain metastasis after whole brain palliative radiotherapy in metastatic renal cell cancer progressing on first-line sunitinib: a case report. World J Oncol.

[18] Mojica CV, Aguas GV, Cornelio GT, Damian LF (2021). Prolonged survival using first-line pazopanib in a Filipino male with renal cell carcinoma and brain metastasis: a case report. Case Rep Oncol.

